# The Clinical Utility of the Chen Internet Addiction Scale—Gaming Version, for Internet Gaming Disorder in the DSM-5 among Young Adults

**DOI:** 10.3390/ijerph16214141

**Published:** 2019-10-28

**Authors:** Chih-Hung Ko, Sue-Huei Chen, Chih-Hung Wang, Wen-Xiang Tsai, Ju-Yu Yen

**Affiliations:** 1 Department of Psychiatry, Kaohsiung Municipal Siaogang Hospital, Kaohsiung Medical University, Kaohsiung City 812, Taiwan; chihhungko@gmail.com; 2 Substance and Behavior Addiction Research Center, Kaohsiung Medical University, Kaohsiung City 807, Taiwan; 3 Department of Psychiatry, Kaohsiung Medical University Hospital, Kaohsiung Medical University, Kaohsiung City 807, Taiwan; 4 Department of Psychology, National Taiwan University, Taipei 10617, Taiwan; shchen@ntu.edu.tw; 5 Department of Guidance and Counseling, National Changhua University of Education, Changhua 50074, Taiwan; ethicgm@gmail.com; 6 Department of Psychology, Kaohsiung Medical University, Kaohsiung City 807, Taiwan; thunderracco@gmail.com; 7 Department of Psychiatry, Kaohsiung Municipal Ta-Tung Hospital, Kaohsiung Medical University, Kaohsiung 801, Taiwan; 8 Department of Psychiatry, Faculty of Medicine, College of Medicine, Kaohsiung Medical University, Kaohsiung City 807, Taiwan

**Keywords:** internet gaming disorder, DSM-5, CIAS, sensitivity, specificity

## Abstract

*Objectives*: The Diagnostic and Statistical Manual of Mental Disorders, Fifth Edition (DSM-5) includes the diagnostic criteria for Internet gaming disorder (IGD). This study evaluated (1) the screening, diagnostic, and prevalence-estimated cutoff points of the Chen Internet Addiction Scale–Gaming Version (CIAS-G) for IGD in the DSM-5; and (2) the differences in the CIAS-G and subscale scores among individuals with IGD, regular gamers (RGs), and other control subjects. *Methods:* We recruited 69 participants with IGD, 69 RGs, and 69 healthy participants based on diagnostic interviews conducted by a psychiatrist according to DSM-5 IGD criteria. All participants completed the CIAS-G and were assessed using the clinical global impression scale. *Results:* The optimal screening and diagnostic cutoff points were 68 or more (sensitivity, 97.1%; specificity, 76.8%) and 72 or more (sensitivity, 85.5%; specificity, 87.0%) for IGD based on DSM-5 criteria, respectively. The 76 or more cutoff point had the highest number needed to misdiagnose and was the optimal prevalence estimated cutoff point. *Conclusions:* The screening cutoff point could be used to identify individuals with IGD for further diagnostic interviewing to confirm the diagnosis in the clinical setting or for two-stage epidemiological evaluation. The diagnostic cutoff point provides a provisional diagnosis of IGD when diagnostic interviewing is unavailable. The prevalence-estimated cutoff point could be used to estimate the prevalence of IGD in large-scale epidemiological investigations when further diagnostic interviewing is impractical. The clinical and epidemiological utility of CIAS-G warrants further study.

## 1. Introduction

Internet gaming disorder (IGD) was included in the *Diagnostic and Statistical Manual of Mental Disorders, Fifth Edition* (DSM-5) because of its public health importance. Its global prevalence ranges between 0.5% and 6% [1,2,3]. Based on clinical evidence and in the interests of public health [4], gaming disorder (GD) was also recently included in the *International Classification of Diseases, Eleventh Revision* (ICD-11) [5]. Many researchers have cautioned against including IGD in the DSM-5 or GD in the ICD-11 because of inadequate scientific evidence [6]. Further research on IGD and GD and their public impacts are necessary to resolve these concerns.

A well-designed epidemiological study with a randomized sample derived from a community population can provide favorable insights into the public importance of a psychiatric disorder [7]. In the DSM-5, it has been suggested that epidemiological studies must be conducted to determine the prevalence of or correlates of IGD [1]. On the one hand, an interview-based epidemiological study can confirm the diagnosis, prevalence, symptom presentation, and demographic characteristics of a disorder. However, the diagnostic approach may be costly and time-consuming. On the other hand, evaluating a psychiatric disorder using a self-reporting questionnaire with high diagnostic accuracy may be more practical in a large-scale investigation.

### 1.1. The Self-Reporting Questionnaire of IGD

Cho et al. developed the Internet Addiction Scale based on DSM-5 IGD criteria [8]. Lemmens developed the polytomous or dichotomous IGD scale with nine items of DSM-5 IGD criteria and determined GD based on latent class analysis [9]. Pontes et al. developed a new nine-item scale (Internet Gaming Disorder Scale–Short Form (IGDS-SF9)) to assess IGD. They demonstrated that the nine items of the IGDS-SF9 are valid and reliable, and these items proved to be highly suitable for measuring IGD [10]. The IGDS-SF9 has been translated into different languages and is widely used in different countries [11]. Individuals who answer “often” and “very often” to all nine questions in this scale attain a score 36 of 45 points and are classified as individuals with GD. However, the diagnostic validity of the cutoff point has not been empirically evaluated. Király developed another widely used measure, the Ten-Item Gaming Disorder Test, which proved to be a valid and reliable instrument for assessing IGD based on DSM-5 criteria [12]. Using the Ten-Item Internet Gaming Disorder Test, Chiu et al. reported that the prevalence of IGD among adolescents was 3.1% [2]. That ass the only study to determine the cutoff point based on diagnostic interviewing. The cutoff point had higher specificity with lower sensitivity as the case number was relatively small in the study sample. A questionnaire with favorable psychometrics validated based on standard criteria, such as DSM-5 IGD criteria or ICD-11 GD criteria, and with a clear cutoff point for diagnosis, is essential for conducting a large-scale epidemiological study of IGD in community populations.

Although both the IGDS-SF9 and the Ten-Item Gaming Disorder Test have excellent psychometric performance, their questions are based on IGD criteria that usually ask two questions in one sentence. For example, it asks, "Do you feel more irritability, anxiety, or even sadness when you try to either reduce or stop your gaming activity?" Furthermore, neither of these tests provide cutoff points based on diagnostic interviewing in adults. On the other hand, [13] reviewed epidemiological studies and explained that differences in methodologies make it difficult to compare previous findings. The Chen Internet Addiction Scale (CIAS) [14] is one of the most popular self-reporting questionnaires on Internet addiction, particular in Asia [15]. It assesses the symptom dimensions of tolerance, withdrawal, and compulsive use and the problem dimensions of interpersonal and health-related problems and time management. The cutoff point of the CIAS has been provided for adolescents and adults [16,17]. The scale was revised to assess online gaming addiction (Chen Internet Addiction Scale—Gaming Version (CIAS-G)) before the DSM-5 criteria had been proposed [18]. Its diagnostic utility should be evaluated, so that the CIAS-G can be used as a measure to assess IGD based on dimensions rather than on questions based on IGD criteria. 

### 1.2. The Cut-Off Point of a Self-Reporting Questionnaire for IGD

A cutoff point with adequate sensitivity can be used to screen individuals with excessive gaming at university or the workplace. Moreover, a cutoff point with high diagnostic accuracy can be used to identify individuals needing help for their uncontrolled gaming. However, using the cutoff point to distinguish individuals with IGD from healthy control individuals with little gaming experience might underestimate the cutoff score. Griffiths suggested that IGD criteria should be evaluated in terms of their ability to distinguish IGD from healthy gamers [19]. Thus, the relevant diagnostic utility of the scale should be evaluated between individuals with IGD and regular gamers (RGs). 

In a massive epidemiological investigation, the cutoff point is usually used to estimate prevalence based on a self-reported scale. However, if the prevalence rate was lower—for example, 10%—the best diagnostic cutoff point determined based on the balance in sensitivity and specificity might inflate the prevalence because the false positive (FP) number was higher than the false negative (FN) number. For example, in a scale with 90% sensitivity and 90% specificity for a disorder with 10% prevalence, the prevalence would be 18%, 9 + 9/100, in an epidemiological investigation. Habibzadeh had developed the “number needed to misdiagnose (NNM)," a measure of diagnostic tests’ effectiveness, to adjust the cut-off point for the misdiagnosing number based on the prevalence of an individual disorder [20]. Thus, to develop the cut-off point to determine the reasonable prevalence might contribute to the research utility of CIAS-G.

The NNM was determined as follows:

Weighted NNM = 1/(C*FN + FP)(1)

C: The cost of an FN is C times the cost of an FP.

### 1.3. The Aims

Furthermore, an ideal scale should adequately represent the severity of IGD. Thus, its score should be correlated with the clinical severity of IGD, particularly among the case group. Therefore, the present study evaluated (1) the best screening, diagnostic, and prevalence-estimated cutoff points of the CIAS-G for differentiating adults with IGD from RGs (without IGD); and (2) the clinical and diagnostic utility of the CIAS-G and subscales for differential IGD from RGs. We demonstrate the recruiting of participants, evaluating of measures, and statistical analysis of the cut-off point in the methods section. Then, the receiver operating characteristic (ROC) curves of the CIAS-G and the diagnostic accuracy of candidate cut-off points are shown in the results section. The discussion section is focused on cut-off points and the utility of CIAS-G and its clinical implications. 

## 2. Methods

Ethical code from IRB (the Institutional Review Board of Kaohsiung Medical University Hospital, Taiwan): KMUHIRB-SV(II)-20150081.

### 2.1. Participants

Participants were recruited based on an age (±3 years) and sex-matched case-control study design. Individuals with IGD (IGD group), RGs (RG group), and non-gamers (control group) were recruited through advertisements on university campuses and online bulletin boards of universities in Taiwan from April 2017 to February 2018. The following inclusion criteria were adopted for the IGD group: (1) age 20–38 years with college education level or higher, (2) online game activity ≥4 hour/day on weekdays and ≥6 hour/day on weekends, and (3) a consistent pattern of Internet gaming for >2 years. Those who met the inclusion criteria participated in interviews to confirm their diagnosis based on IGD criteria defined in the DSM-5. Those who fulfilled the DSM-5 criteria were classified as the IGD group.

The recruitment criteria of the control group were that their nonessential Internet use was <4 hours/day and that they did not perform regular gaming. RGs participated in regular online gaming (≥3 d/week) without fulfilling the diagnostic criteria of IGD. The diagnosis of both groups was confirmed through psychiatric interviews.

In total, 207 participants, i.e., 69 in each of the three study groups, were included after informed consent was obtained. This study was approved by the Institutional Review Board of Kaohsiung Medical University Hospital, Taiwan.

### 2.2. Measures

#### 2.2.1. DSM-5 Diagnostic Criteria for IGD

We developed a semi-structured interview schedule to examine the severity and frequency of each DSM-5 criterion for IGD. Participants fulfilling five or more criteria were included in the IGD group.

#### 2.2.2. CIAS-G

The CIAS is a 4-point, 26-item self-report scale assessing five dimensions of Internet addiction: compulsive use, withdrawal, tolerance, problems with interpersonal relationships, and problems with health and time management [14]. The colloquial expressions of the CIAS were modified to assess participants’ online gaming experiences (CIAS-G), with a Cronbach’s α of 0.96 [18]. The total CIAS-G score ranges from 26 to 104. A higher CIAS-G score indicates a higher severity of IGD. The scores for the symptoms of compulsive use (score ranging from 5 to 20), withdrawal (score ranging from 5 to 20), and tolerance (score ranging from 4 to 16) were summed, which represented the core Internet addiction symptoms. The scores of problems with interpersonal relationships (score ranging from 7 to 28) and problems with health/time management (score ranging from 5 to 20) were summed, which represented Internet addiction-related problems.

#### 2.2.3. The Clinical Global Impression Scale (CGI)

The CGI [21] was modified for the scoring interpretation of IGD [22]. We used the following CGI question for IGD: “Considering your total clinical experience with this particular population, how mentally ill is the patient at this time?” The scores for this question were generally interpreted as follows by a psychiatrist based on information gathered in psychiatric interviewing:

- 1 = Normal; not at all ill.
- 2 = Excessive Internet gaming without fulfilling the IGD criteria.
- 3 = Fulfilling IGD criteria with mild, limited, or short-term functional impairment.
- 4 = Functional impairment is moderate in one field, such as academic, social, work, or health.
- 5 = Moderate functional impairment in multiple dimensions.
- 6 = Severe impairment in one field.
- 7 = Severe impairment in multiple dimensions of daily life function.

This modified CGI scale was used to represent IGD severity with scores ranging from 1 to 7 in this study.

### 2.3. Statistical Analysis

A series of one-way analyses of variance (ANOVAs) were conducted to evaluate differences in CIAS-G total and subscale scores among the IGD, RG, and control groups based on DSM-5 criteria. Their differential performance of IGD was tested by receiver operating characteristic (ROC) curves and diagnostic accuracy. The sensitivity, specificity, positive predictive value, negative predictive value, diagnostic accuracy, and Cohen’s κ value of the indicated cutoff points of the CIAS-G to differentiate IGD from regular gaming were evaluated to determine the optimal cutoff point of the CIAS-G. The ROC curves of the CIAS-G were determined with reference to the diagnostic criteria of IGD. 

The screening cutoff point with (1) sensitivity higher than 85%, (2) specificity higher than 70%, and (3) the highest Cohen’s κ value was determined as the optimal cutoff point. The optimal diagnostic cutoff point was determined based on the optimal trade-off point between sensitivity and specificity [23]. Lastly, the estimate cutoff point for determining prevalence was determined based on NNM. It determined the best cutoff point to have the maximal proportion of the population to classified into the correct group. Based on our previous epidemiological study with the CIAS scale, the prevalence of IGD was assumed to be 10.8% [24]. Further, as the comparison group in this study contained regular gamers, but not the general population, the (1-Prevalence) should be adjusted to the prevalence of regular gamer. Wu et al had reported that 47.3% of the adult population plays games [3]. Based on that data, participants without regular gaming were not likely to classify to be IGD based their scoring of CIAS-G. Thus, we adjusted the NNM to be:

Weighted NNM = 1/(C*FN + FP);(2)

The *t*-tests were conducted to evaluate the difference in CIAS-G total and subscale scores between low and high-CGI subgroups in the IGD group to demonstrate the performance of CIAS-G at determining the severity of IGD. A *p*-value of <0.05 was considered significant for all analyses, which were performed using SPSS version 14.0 software (SPSS, Chicago, IL, USA).

## 3. Results

A total of 69 participants were classified into the IGD group. Sixty-nine healthy controls without regular gaming and 69 RGs were classified as nongaming control subjects and the RG group, respectively. Female participants comprised 21.7% of each group (Table 1).

### 3.1. Discriminatory Validity and Utility of CIAS-G for Differential IGD in DSM-5

The results in Table 1 demonstrated a significant difference in subscale scores of compulsive use, withdrawal, tolerance, problems with interpersonal relationships, and problems with health and time management among the IGD, RG, and nongaming control groups. These results demonstrated that the IGD group had higher scores than the RG group, and that the RG group had higher scores than control subjects. Thus, the CIAS-G could adequately discriminate the IGD group from the RG and control groups.

**Figure 1:**
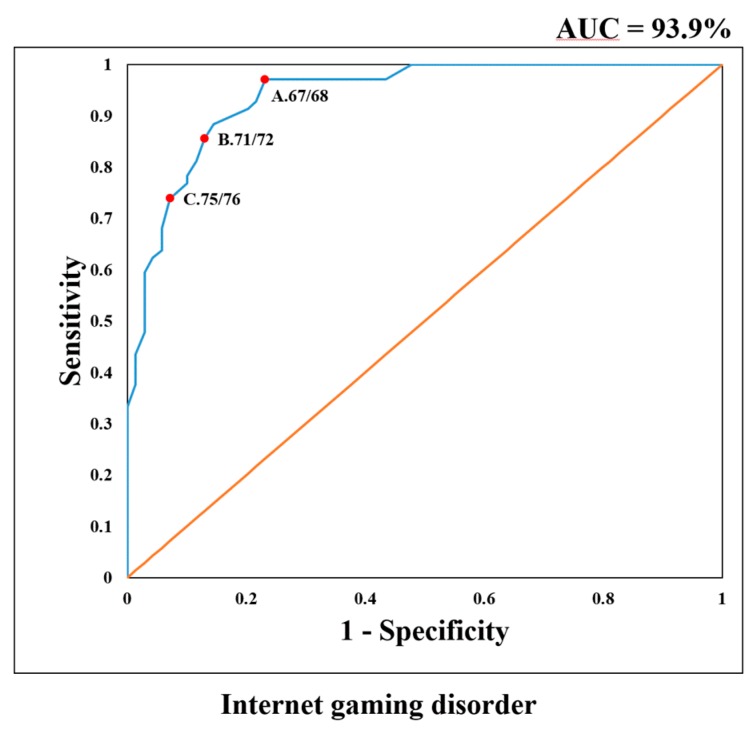
Results of the area under the receiver operating characteristic curve analysis for the Chen Internet Addiction Scale score (score ranges from 26 to 104) for classifying Internet gaming disorder. A: the screening cutoff point; B: the diagnostic cutoff point; C: the prevalence estimated cutoff point.

The CIAS scores of all control participants were lower than the cutoff point (68 or more) for Internet addiction in a previous study [17]. We determined the optimal cutoff point to distinguish the IGD group from the RG group based on Cohen’s κ value and ROC curve. The area under the curve (AUC) of the ROC was 93.9%, demonstrating the high diagnostic performance of the CIAS-G for IGD (Figure 1). The evaluation results provided in Table 2 demonstrate that a score of 68 or more had a higher sensitivity of 97.1% and a lower specificity of 76.8%, and this score was the optimal screening cutoff point with the highest Cohen’s κ value. 

A cutoff point of 72 or more had sensitivity of 85.5%, specificity of 87.0%, and positive predictive value of 86.8%. It was the optimal diagnostic cutoff point of the CIAS-G for IGD. It provided the best cutoff point to determine the IGD classification for an individual with regular gaming.

The NNM analysis demonstrated that one out of every 11.4 and 15.8 individuals would be misdiagnosed based on 68 and 72 cutoff points, respectively. The Table 2 also demonstrated that one out of every 18.4 individuals would be misclassified in the general population based on a cut-off point of 76. Thus, 76 or more is the optimal prevalence-estimate cut-off point of CIAS-G for epidemiological evaluation.

### 3.2. Discriminative Ability for the Severity of IGD

Within-group analysis demonstrated that the CGI score estimated by psychiatrists was correlated with scores for tolerance (*r* = 0.26, *p* = 0.033), withdrawal (*r* = 0.28, *p* = 0.018), time management problems (*r* = 0.33, *p* = 0.006), and interpersonal and health-related problems (*r* = 0.26, *p* = 0.032). We then classified the subjects with CGI scores higher than four as the group with severe IGD. The further evaluation results in Table 3 demonstrated that the severe group had higher CIAS-G subscale and total scores, except for withdrawal symptoms. Thus, the CIAS-G total score and subscale scores of tolerance, interpersonal health-related problems, and time management problems could adequately discriminate the severity of IGD.

## 4. Discussion

Kuss et al. suggested that gold standard criteria and a conclusive scale cutoff point contribute to the appropriate assessment of prevalence and enable the comparison of different studies [25]. The present study evaluated the optimal cutoff point of the CIAS-G, a popular scale in Asia [15], based on psychiatrist-performed diagnostic interviewing to differentiate DSM-5 IGD from regular gaming. The results provided the screening cutoff point, optimal diagnosing cutoff point, and prevalence-estimated cutoff point to contribute to the clinical and research utility of the CIAS-G.

### 4.1. Cutoff Point and Utility of CIAS-G for IGD in DSM-5

Our results demonstrated a higher AUC (93.9%) in ROC analysis for differentiating IGD from RG. The multiple dimensions of the CIAS-G could represent IGD severity and contribute to the high diagnostic performance of CIAS-G in ROC analysis. The screening cutoff point of IGD was 68, with a high sensitivity of 97.1% and an acceptable specificity of 76.8%. Thus, based on this cutoff point, 97.1% of individuals with IGD could be effectively screened based on this cutoff point in the clinical setting.

The cutoff point could also be used in epidemiological studies when interviewing resources are limited. In the general population, the number of RGs without IGD is much higher than that of individuals with IGD. The false-positive rate of 68 cutoff points (19.3%) will be increased in an epidemiological study conducted in the general population. Thus, candidates reaching the cutoff point should be interviewed by a psychiatrist to confirm their diagnosis in two-stage epidemiological studies.

The present study demonstrated the 72 is the best cutoff point to diagnose individuals of IGD from RGs. In this study, the number of participants in the IGD and RG groups was kept equal to calculate sensitivity and specificity. The CIAS-G has been evaluated for the multiple dimensions of IGD and provides varied information. Furthermore, the large range of scores of the CIAS-G makes it possible to select a cutoff point with balanced performance in sensitivity and specificity. The optimal trade-off between sensitivity and specificity of the 72 cutoff point made it suitable to evaluate IGD among individuals with regular gaming. The 72 cutoff point provides a good positive predictive rate of 86.8% and a negative predictive rate of 85.7%. It could provide an alternative tool to identify individuals with IGD when diagnostic interviewing is unavailable. However, 14.5% of IGD individuals and 13% of regular gamers will be misdiagnosed. As the proportion of regular gamers is higher than that of IGD, the prevalence will inflate when the 72 cutoff point is used in an epidemiological study. Thus, the prevalence rate determined by 72 shall be adjusted based on the estimated prevalence rate and proportion of regular gamers.

Previous studies have suggested 64 and 68 as cutoff points for Internet addiction among adolescents [16] and college students [17], respectively. In those previous studies, control subjects included those who did not use the Internet frequently and who scored very low on the CIAS. Because nongaming individuals are not likely to be candidates for IGD in the clinical setting, we evaluated the cutoff point for differentiating individuals with IGD from RGs who scored higher on the CIAS-G than nongaming control subjects. This made the cutoff point more reasonable and higher than those in previous studies. Furthermore, Tateno et al. found that the mean score of college students had reached the screening cutoff point of the Young’s Internet Addiction Test [26] 20 years after the test was developed. The cutoff point should be revised to keep update the validity for gaming behavior, which is rapid shifting in modern society.

The present study determined the prevalence estimated cutoff point based on NNM. The NNM determines the cutoff point based on the lowest misdiagnosing proportion. Under assuming an IGD prevalence of 10.8% and regular gaming prevalence of 47.3%, 76 is the best cutoff point with the lowest number of misclassifications in epidemiological evaluation. Thus, the prevalence of IGD could be determined by 76 cutoff points of CIAS-G under a similar prevalence and gamer proportion. However, if the prevalence and gamer proportion were to be different from this study, the NNM could be adjusted based on the sensitivity and specificity of the cut-off point from 67–77, shown in Table 2. (The NNM = 1/[prevalence*(1-Sensitivity) + (gamer proportion − prevalence)%*(1 − Specificity)]). Without adjustment, most studies will inflate the prevalence of IGD, as FP is much higher than FN, particularly in disorders with lower prevalence, such as IGD.

### 4.2. Severity of IGD or GD Represented by CIAS-G

In this study, the CIAS-G total and subscale scores could significantly differentiate among the IGD, RG, and control groups. The results suggested that each dimension of the CIAS-G could differentiate IGD severity among these three groups. They also demonstrated that the score of the RG group was close to the mean score of the IGD and nongaming control subjects. The results indicated that the RG group showed some symptoms of IGD, although the symptoms did not reach the threshold of IGD. In the IGD group, the CIAS-G total and subscale scores, except for withdrawal symptoms, between the high and low-CGI groups, were different. The CIAS-G and its subscales could differentiate severity levels in the IGD group.

### 4.3. Clinical Implications

The CIAS-G had different cutoff points for DSM-5 IGD and HG. The 68 cutoff point was the screening cutoff point for IGD. Thus, adults with CIAS-G scores higher than 67 should be diagnostically interviewed to determine whether they fulfill the criteria of IGD in the DSM-5. Adults with CIAS-G scoring 72 or more, which is the optimal diagnostic cutoff point of IGD, should be referred to a psychiatrist to confirm the diagnosis, and if necessary, a brief intervention should be provided to increase their motivation to change. Besides, cognitive behavioral therapy [27] should be provided for treating IGD. Furthermore, psychiatric comorbidities should be evaluated and treated. The CGI scale should be used to assess individuals with clinical diagnoses to determine severity based on functional impairment. The score can be used for the follow-up of treatment effect and to evaluate its prediction in the outcome.

The previous study demonstrated a higher prevalence of IGD in the Asia area [28]. The cut-off points in the current study could be utilized to identify well, an individual with a risk of IGD in Asian areas. However, the prevalence rate is relatively lower in European areas (1.16%) [29]. Based on the formula of NNM, the prevalence-estimated cut-off point in Europe should be adjusted based on its prevalence. Further, the cultural effects could shape the presentation of addictive and problematic gaming [30]. To evaluate IGD by using CIAS-G in European or other areas, could provide a comparison in severity and estimated prevalence between Asia and other areas.

## 5. Limitations

First, the diagnosis of IGD was reached solely based on participants’ responses in a psychiatric interview. Further information collected from other sources, such as parents or partners, may be required to strengthen diagnoses. Second, recall bias may not be excluded without direct observation of gaming behaviors. Third, the prevalence and gaming proportion were referred to the previous study for the calculation of NNM. A directly epidemiological study was necessary to provide precise calculations. Lastly, we did not estimate the sample size before recruiting participants. We recruited as many participants with IGD, who were RGs, and who were non-gamers as possible in limited duration. A larger sample size, particularly of participants scoring from 66 to 77 in CIAS-G, might provide more information to grant an exact determination of the cut-off point. However, these are highly time and resource-consuming. 

## 6. Conclusions

The CIAS-G and its subscales could differentiate the IGD from RGs and nongaming individuals. Except for the withdrawal subscale, they could determine the severity level in individuals with IGD. On the basis of an evaluation of sensitivity and specificity for differentiating DSM-5 IGD from regular gaming, the 68 and 72 cutoff points are the screening and optimal diagnostic cutoff points, respectively, for IGD in the DSM-5. These cutoff points could be used to screen and identify people who need individualized help for their uncontrolled gaming. Seventy-six was the optimal cutoff point to determine the prevalence in an epidemiological study based on CAIS-G. These implications should be evaluated in future intervention and epidemiological studies.

## Figures and Tables

**Table 1:** Characteristics of Internet gaming disorder (IGD), regular gamer (RG), and nongaming control groups.

	IGD *n* (%)	RG *n* (%)	Control *n* (%)	*χ^2^*	
Gender					
Male	54 (78.3)	54 (78.3)	54 (78.3)	0.00	
Female	15 (21.7)	15 (21.7)	15 (21.7)		
	**Mean (SD)**	**Mean (SD)**	**Mean (SD)**	***F***	**Post hoc**
Age (years)	25.32 (4.20)	24.59 (3.41)	26.87 (3.82)	6.379 **	Control > RG
CGI score	4.52 (0.93)	1.39 (0.49)	1.00(0.0)	693.248 ***	IGD > RG > Control
CIAS-G total score	82.87 (10.49)	55.87 (14.69)	30.86 (8.11)	357.581 ***	IGD > RG > Control
Tolerance symptoms	12.68(2.11)	8.65 (2.51)	4.72 (1.34)	260.750 ***	IGD > RG > Control
Withdrawal symptoms	16.16 (2.29)	11.67 (3.22)	5.99 (1.79)	286.407 ***	IGD > RG > Control
Compulsive symptoms	15.51 (2.40)	9.84 (3.15)	5.74 (1.83)	261.869 ***	IGD > RG > Control
Time management problems	16.75 (2.44)	11.29 (3.50)	6.06 (1.85)	274.122 ***	IGD > RG > Control
Interpersonal and health problems	21.77 (3.62)	14.39 (4.19)	8.35 (2.61)	249.716 ***	IGD >RG> Control

CGI: Clinical Global Impression Scale; CIAS-G: Chen Internet Addiction Scale—Gaming Version. ** *p* < 0.01; *** *p* < 0.001.

**Table 2:** Optimal cutoff point scores for DSM-5 Internet gaming disorder of the Chen Internet Addiction Scale—Gaming Version.

	Sensitivity (%)	Specificity (%)	PPR (%)	NPR (%)	DA (%)	Cohen’s Kappa	NNM
IGD versus RG							
CIAS-G							
66/67	97.1	75.4	79.8	96.3	86.3	0.73 ***	10.76
**67/68**	**97.1**	**76.8**	**80.7**	**96.4**	**87.0**	0.74 ***	**11.39**
68/69	92.8	78.3	81.0	91.5	85.5	0.71 ***	11.50
69/70	91.3	79.7	81.8	90.2	85.6	0.71 ***	11.98
70/71	88.4	85.5	85.9	88.1	87.0	0.74 ***	15.28
**71/72**	**85.5**	**87.0**	**86.8**	**85.7**	**86.3**	0.73 ***	**15.85**
72/73	81.2	88.4	87.5	82.4	84.8	0.70 ***	15.96
73/74	78.3	89.9	88.5	80.5	84.0	0.68 ***	16.58
74/75	76.8	89.9	88.3	79.5	83.3	0.67 ***	16.15
**75/76**	**73.9**	**92.8**	**91.1**	**78.0**	**83.4**	0.67 ***	**18.36**
76/77	68.1	94.2	92.2	74.7	81.2	0.62 ***	17.98

CIAS-G: Chen Internet Addiction Scale—Gaming Version; DA: diagnostic accuracy; NPR: negative predictive rate; PPR: positive predictive rate. NNM: Number needed to misdiagnose *** *p* < 0.001.

**Table 3:** Difference in Chen Internet Addiction Scale—Gaming Version between high and low Internet gaming disorder(IGD) severity.

The Score in CIAS-G	High Severity *N* = 37 Mean (SD)	Low Severity *N* = 32 Mean (SD)	*t*
CIAS-G total score	86.49(9.99)	78.69(9.59)	−3.29 **
Tolerance symptoms	13.35(2.07)	11.91(1.92)	−2.99 **
Withdrawal symptoms	16.62(2.36)	15.63(2.10)	−1.84
Compulsive symptoms	16.24(2.22)	14.66(2.35)	−2.88 **
Time management problems	17.57(2.02)	15.81(2.57)	−3.17 **
Interpersonal and health problems	22.70(3.68)	20.69(3.29)	−2.38 **

High severity of IGD: IGD subjects with clinical global impression (CGI) scores of five or more; low severity of IGD: IGD subjects with CGI scores of four or lower. ** *p* < 0.01.
